# Serological Evidence of Hepatitis B and E and Dengue Coinfection in Chadian Patients and Impact on Lipidemia Profile

**DOI:** 10.1155/2022/8373061

**Published:** 2022-09-16

**Authors:** Alexandre Kanga Djasrabe, Borris Rosnay Tietcheu Galani, Moussa Mahamat Ali, Fissou Henry Yandai, Bessimbaye Nadlaou, Mayann Habkreo, Nicolas Yanou Njintang

**Affiliations:** ^1^Laboratory of Applied Biochemistry, Department of Biological Sciences, Faculty of Science, University of Ngaoundere, Ngaoundere, Cameroon; ^2^Department of Gastroenterology and Internal Medicine, National Reference University Hospital of N'Djamena, N'Djamena, Chad; ^3^Laboratoire Mobile Des Virus Hémorragiques et Respiratoires, N'Djamena, Chad; ^4^Laboratories Department, National Reference University Hospital of N'Djamena, N'Djamena, Chad

## Abstract

**Objective:**

Viral hepatitis is an endemic disease in Chad. However, few studies have documented coinfection cases and their impact on cardiovascular risk. This study is aimed at analyzing hepatitis B, E and dengue coinfection in a Chadian cohort and gauge its effect on lipidemia. *Patients and Methods*. From February to May 2021, 179 subjects were recruited from the Department of Gastroenterology and Internal Medicine of the National Reference University Hospital of N'Djamena and tested for viral hepatitis markers, including HBsAg and IgM/IgG anti-HEV and dengue infection, using the NS1/IgM/IgG kit. Serum transaminases and biomarkers of lipid profiles were assayed by colorimetry, and atherogenic indexes (AI) and coronary risk (CRI) were calculated.

**Results:**

Of the 179 subjects surveyed, 21.22% (38/179) tested positive for hepatitis B, 20% (27/135) for hepatitis E, and 1.66% (2/120) for dengue. However, most of the patients were found to be asymptomatic. Hepatitis B/E coinfection was more frequent in the study population (5.02%; 9/179) than dengue/hepatitis E coinfection (0.83%; 1/120; IgM). The prevalence of anti-HEV IgG antibodies was higher (18.52%) than that of IgM (1.48%). Furthermore, IgG antibodies levels in HEV-monoinfected subjects (11.05 ± 1.93 IU/mL, *N* = 15) were significantly higher (*p* < 0.05) than in coinfected patients (5.40 ± 1.31 IU/mL, *N* = 9). Subjects coinfected with HEV/HBV were associated with a significantly higher risk of lipodystrophy (coronary risk: 88.89% vs. 35.3%, relative risk (RR) = 2.55; *p* = 0.014), than HEV-monoinfected subjects, as evidenced by higher mean levels of triglycerides levels (219.88 ± 14.67 mg/dL vs. 191.82 ± 4.66  mg/dL, *p* < 0.05), more reduced HDL-C levels (9.05 ± 1.62 mg/dL vs. 18.93 ± 2.35 mg/dL, *p* < 0.05), increased mean CRI (13.81 ± 2.39 vs. 6.89 ± 1.93, *p* < 0.01), and AI (1.46 ± 0.10 vs. 1.05 ± 0.05, *p* < 0.01) values. However, they had normal transaminase values and a lower risk of developing a liver injury, although not significant (alanine aminotransferase: 0% vs. 29.4%, RR = 1, *p* = 0.128; aspartate aminotransferase: 0% vs. 5.88%, *p* = 1) than this group.

**Conclusion:**

HBV/HEV coinfection is frequent in the Chadian cohort and associated with an important risk of dyslipidemia. Further research is required to elucidate the mechanism of action.

## 1. Introduction

Viral hepatitis is a major health threat around the world. According to the World Health Organization (WHO), approximately 1.34 million deaths were recorded in 2015, mostly due to cirrhosis and primary liver cancer caused by viral hepatitis [1]. Globally, more than 257 million people live with chronic hepatitis B infection, while 20 million are infected with hepatitis E, with Pacific and African regions being the most affected areas [1]. Unfortunately, many infected patients are unaware of their status, and some structural barriers combined with stigmatization and social discrimination contribute to worsening the vulnerability of these patients and their access to health care services in Africa [2].

In Chad, viral hepatitis B and E are highly endemic and are often detected during routine blood donation examinations [3, 4]. In the N'Djamena city located in the Center-West of Chad, previous epidemiological studies have documented a seroprevalence of 16.1% for hepatitis B s (HBs) antigen in HIV-infected people [5] and 41% for HBc antibodies in 1106 seronegative blood donors recruited at the national Blood Transfusion Centre [4]. Other studies suggest that approximately 2% of patients with seropositive hepatitis B in this locality have the HBe antigen as a marker of chronicity [6]. However, for hepatitis E, limited data are available. In addition, hepatitis B/E coinfection has been poorly explored. An earlier retrospective study by Coursaget et al. reported seroprevalences of 22% for acute hepatitis B, 66% for acute hepatitis E, and 10% for dual acute hepatitis B and E infections in 41 patients referred to the Central Hospital of N'Djamena [7].

Furthermore, increasing evidence suggests that complications in viral hepatitis may arise from superinfection with hepatic viruses [8] or hemorrhagic fever viruses such as the dengue virus (DENV), which is a primary cause of acute hepatitis [9]. This condition favors liver fibrosis and increases the progression to hepatocellular carcinoma and liver failure [10]. Sporadic cases of dengue/hepatitis B [11] and dengue/hepatitis E [12] coinfections were detected in China and India, respectively. However, no information on the status of this coinfection is available in Chad. Likewise, its effects on metabolic parameters remain poorly understood. However, dengue and viral hepatitis can induce alterations in serum lipid levels [13, 14].

The liver is a central organ involved in the sequestration, remodeling, and redistribution of lipid metabolites, including low-density lipoproteins transporting cholesterol (LDL-C) and high-density lipoproteins transporting cholesterol (HDL-C), triglycerides (TGs), and total cholesterol (TC). Increased LDL-C has been shown to increase plasma cholesterol, raising the risk of cardiovascular and coronary heart disease [15].

Several studies have demonstrated a link between dyslipidemia and viral hepatitis by showing that hepatic dysfunction is associated with hepatitis B and E can interfere with lipid metabolism in vivo and modify plasma lipid and lipoprotein patterns [14, 16]. While some reports indicate that asymptomatic hepatitis B positive carriers may be associated with a low risk of dyslipidemia compared to healthy controls [17], others found hyperlipidemia in the hepatitis B group as evidenced by high TC, LDL-C, and TG levels [14, 16]. However, the effect of hepatitis B/E coinfection on lipid profiles remains unknown. Nevertheless, some studies have shown that HCV/HIV coinfection protects HIV patients from lipoprotein-induced atherogenic abnormalities [18, 19]. Therefore, the impact of co-infection on serum lipids should be evaluated for other hepatic viruses.

This study aimed to contribute to the epidemiological profile of viral hepatitis in Chad by 1) assessing the overall hepatitis B and E and DENV prevalence in the target population in N'Djamena as well as the impact of socio-demographic factors on this prevalence; 2) determining the levels of hepatitis B/E coinfection, and superinfection with DENV and 3) analyzing the impact of coinfection on transaminase values and lipid parameters and identifying the cardiovascular and coronary risk.

## 2. Materials and Methods

### 2.1. Study Site

This study was conducted at the National Reference University Hospital (NR-UH) of N'Djamena in Chad. N'Djamena is the capital and largest city of the country, located in the center-west region at latitude 12°06′36^″^N and longitude 15° 03′00′′E, at the confluence of the Chari and Logon rivers. Its population is estimated to be 1,476,115 inhabitants according to recent data from the prospects of the United Nations World Organization [20]. The climate is a hot-semi-arid type with a short-wet season (June-September) and a lengthy dry season (October-May).

### 2.2. Study Design and Participants

A cross-sectional and analytical study was carried out from February 22 to May 20, 2021, at the Department of Gastroenterology and Internal Medicine of the NR-UH of N'Djamena. One hundred and seventy-nine (179) participants were first administered a well-structured questionnaire to record sociodemographic parameters. The sample size was calculated using the Lorentz formula as described elsewhere [21]. The reference prevalence used was 13.5% reported by Bessimbaye et al. [5] in N'Djamena. All participants aged at least 18 years, with no antecedent of vaccination against hepatitis B, with no signs of dyslipidemia were included. However, individuals with diabetes, alcoholics, those with liver complications, and those previously vaccinated against hepatitis B were excluded.

### 2.3. Ethical Considerations

This study was carried out according to the principles of the Declaration of Helsinki. Institutional ethical clearance was obtained from the Chad National Bioethics Committee under the reference number N° 002/PR/MESRI/DG/CNBT/2021. The Director of the NR-UH granted permission for sample collection (Authorization N°107/MSP/SE/DG/CHU/-RN/DC/2021). The objectives, benefits, and risks were clearly explained in French and Arabic to all participants before their inclusion in the study. Written and signed informed consent was obtained from all participants prior to blood collection.

### 2.4. Sample Collection and Laboratory Analysis

Consenting patients and fasting for at least 12 h were collected in two tubes. Four milliliters of blood sample were placed in a dry tube. After collection, all blood samples were centrifuged at 2000 rpm for 5 min, and sera were collected for screening HBV, HEV, DENV, and biochemical parameters.

### 2.5. Determination of HBV Status

Surface antigen (HBsAg) was detected in serum samples using the Abbott kit (Determine™ HBsAg 2) provided by Alere Medical Co. Ltd (Chiba, Japan). Briefly, after adding 50 *μ*L of the sample to the sample pad, the specimen was mixed with biotinylated anti-HBsAg antibodies and black particles coated with these antibodies. The mixture then migrated along with the solid phase with the immobilized avidin on the patient's bar. When HBsAg was present, a black bar appeared on the test strip after 15-20 min. If not present, no black bar was formed on the test strip. To ensure the validity of the test, a red control bar should appear in the control strip after sample migration.

### 2.6. Determination of HEV Status

The screening of hepatitis E virus (HEV) was carried out by detecting anti-HEV IgG/IgM antibodies in serum samples using ELISA kits provided by EUROIMMUN Medizinische Labordiagnostika AG (Lübeck, Germany). The ELISA kit was based on recombinant target antigens of hepatitis E virus genotypes 1 and 3. The results were semiquantitatively evaluated by calculating the ratio of the extinction value of the control or patient sample to the extinction value of calibrator 3 (1 IU/mL, IgG). Then, the results were interpreted as negative for ratio < 0.8, positive for ratio > 1.1, and borderline for ratio > 0.8 to <1.1.

### 2.7. Determination of DENV Status

Dengue diagnosis was carried out using the combined NS1/IgG/IgM kit from Medsinglong Global Group Limited Co. Ltd. (Guangzhou, China) as previously described [22].

### 2.8. Lipid Profile Analysis and Transaminase Activity

Alanine aminotransferase (ALT) and aspartate aminotransferase (AST) activities, total cholesterol (TC), high-density lipoprotein cholesterol (HDL-C), low-density lipoprotein cholesterol (LDL-C), and triglycerides (TG) levels were quantified in serum samples using the BioSystems kits (Barcelona, Spain) following the manufacturer's instructions. The atherogenic (AI) and coronary risk (CRI) indexes were calculated using the following formulas: AI = log (TG/HDL‐C) [23] and CRI = TC/HDL‐C [23, 24].

### 2.9. Statistical Analysis

Data were analyzed using GraphPad Prism 5.0.3 (San Diego, USA). The Chi-square test or Fisher test was used to look for associations between viral hepatitis and the sociodemographic factors of the study population. IgG titers were compared between the HEV-monoinfected and HEV/HBV-coinfected groups using the unpaired Student *t*-test. To gauge the impact of monoinfection and coinfection on parameters of liver function parameters, mean TC, HDL-C, LDL-C, TG, AST, and ALT were compared between study groups. The nonparametric Kruskal-Wallis test followed by Dunn's multiple comparison test was used to examine the mean differences between groups. The probability threshold was set at 5% (*p* < 0.05).

## 3. Results

### 3.1. Sociodemographic Characteristics of the Study Population

Table 1 presents the sociodemographic characteristics of the study population according to sex, age group, profession, marital status, level of education, knowledge of serological status and age, and the district of origin.

It can be seen from this table that the study population is predominantly made up of men (63%) than women (37%). In addition, this population is also dominated by young subjects, in particular people aged 18–25 years and 26–32 years, who each represent 26% of our sample. Similarly, we note that the average age of this population is around 35.03 ± 1.24 years. Regarding the professional situation, the majority of the participants were students (27%) and housewives (23%). On the contrary, farmers made up only 6% of the population. Regarding marital status, most of our participants were composed of married subjects (63%) than single subjects (37%). Regarding the level of study, most of the subjects were at university level (41%), compared to 26% for primary and 21% for secondary. However, very few people (15%) knew their HBsAg status. When we group the participants according to their district of origin, we realize that the majority came from the 7th district (22%), followed by the 10th district (16%).

### 3.2. Seroprevalence of Hepatitis B and E and Dengue

**Figure 1:**
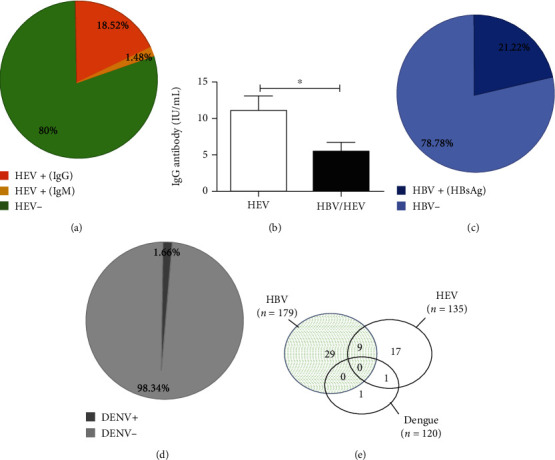
Seroprevalence data in the study population. (a) HEV seroprevalence and (b) anti-HEV IgG titers (IU/mL) compared between mono- and coinfected groups using the student unpaired *t*-test. ^∗^Significant at *p* < 0.05. (c) Seroprevalence of HBsAg. (d) DENV seroprevalence. (e) Venn diagram showing frequencies of monoinfection and coinfection hepatitis B and E and dengue in the study population.

Figure 1 shows the seroprevalence of hepatitis B and E and dengue in the study population. It emerges that hepatitis E and B were more frequent in the surveyed population with a seroprevalence of 20% (Figure 1(a)) and 21.22% (Figure 1(c)), respectively, in contrast to dengue (1.66%, Figure 1(d)). Regarding hepatitis E, the prevalence of anti-HEV IgG antibodies (indicative of an old infection) was higher (18.52%) than IgM (1.48%; indicative of a recent infection) (Figure 1(a)). Moreover, the concentration of anti-HEV IgG antibodies in monoinfected subjects (11.05 ± 1.93 IU/mL, *N* = 15) was significantly greater (*p* < 0.05) than in coinfected patients (5.40 ± 1.31 IU/mL, *N* = 9) as shown in Figure 1(b).

The frequencies of the different cases of coinfection and monoinfection within the surveyed population were assessed using the Venn diagram (Figure 1(e)). It emerges that hepatitis B monoinfection (29/179, 16.2%) was quantitatively more important than hepatitis E (17/135, 12.59%) and dengue (1/120, 0.83%) monoinfection, respectively. A dual hepatitis B and E coinfection was observed (9/179, 5.02%) followed by HEV/dengue coinfection (1/120, 0.83%). However, no hepatitis B/dengue or triple coinfection was observed.

### 3.3. Effect of Sociodemographic Factors on the Seroprevalence of Hepatitis B and E and Dengue

To identify factors that could increase the susceptibility to viral hepatitis, the statistical association between various sociodemographic factors and seropositivity to hepatitis B and E was evaluated.

According to Table 2, gender is statistically associated with hepatitis B. Men were significantly more exposed than women (27.02% vs. 11.76%, RR = 2.297, 95%CI = 1.159-4.717, *p* = 0.0153). Similarly, hepatitis B was statistically correlated with the age of the participants, as well as with the district of origin. Depending on age, subjects of 33–39 (35.48% (11/31), RR = 5.441, 95%CI = 1.797-17.11, *p* = 0.002), 47-53 (31.25% (5/16), RR = 4.792, 95%CI = 1.376-16.43, *p* = 0.022), and 54-60 years (50% (6/12), RR = 7.667, 95%CI = 2.357-24.64, *p* = 0.0014) were significantly more exposed to hepatitis B than subjects aged 18–25 years (6.52%, RR = 1). Regarding the district of origin, the participants in the 6^th^ arrondissement were significantly (33.33% (7/21), RR = 4.5, 95%CI = 1.199-17.87, *p* = 0.0306) more exposed to hepatitis B than the participants from the 8^th^ arrondissement (7.40% (2/27), RR = 1) used as a reference due to their lowest frequency. On the other hand, professional and marital status, level of education, and knowledge of HBV serological status are not statistically (*p* > 0.05) associated with hepatitis B.

As far as hepatitis E is concerned, a statistical association was also noted with sex as shown in Table 3. In fact, men were significantly more exposed than women (27.90% (24/86) vs. 6.12% (3/49), RR = 4.56, 95%CI = 1.59-13.84, *p* = 0.0018). Likewise, hepatitis E is statistically associated with work status. Based on this situation, farmers (42.86% (3/7), RR = 13.71, 95%CI = 2.51-86.62, *p* = 0.014), students (26.31% (10/38), RR = 8.421, 95%CI = 1.533-49.92), *p* = 0.0087), self-employed (26.31% (5/19), RR = 8.421, 95%CI = 1.42-52.22, *p* = 0.022), and traders (25% (4/16), RR = 8, 95%CI = 1.301-50.68, *p* = 0.0366) were significantly more exposed to hepatitis E than housewives (3.12% (1/32), *RR* = 1). On the other hand, age, marital status, level of education, and district of origin are not statistically (*p* > 0.05) correlated with hepatitis E.

### 3.4. Impact of Coinfection and Monoinfection on the Lipid Profile Parameters and Transaminase Activity

**Figure 2:**
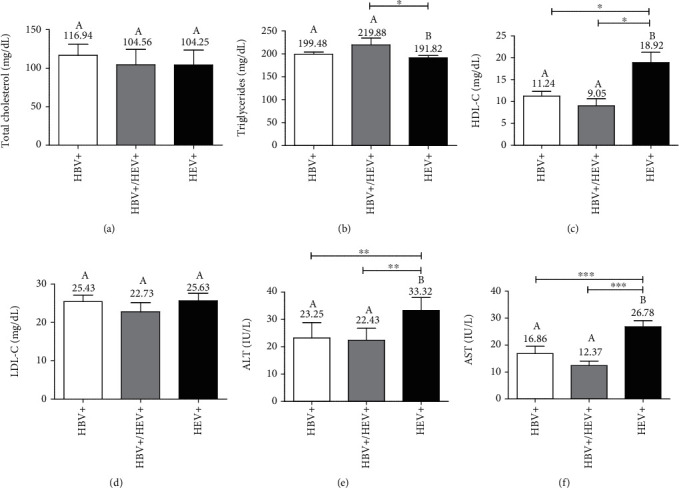
Mean cholesterol, triglycerides, and transaminase levels between HBV/HEV-coinfected and monoinfected subjects. (a) Total cholesterol levels. (b) Triglycerides levels. (c) HDL-cholesterol (HDL-C). (d) LDL-cholesterol (LDL-C). (e) Alanine aminotransferase (ALT) levels. (f) Aspartate aminotransferase (AST) levels. Comparison was carried out using the nonparametric Kruskal-Wallis test followed by Dunn's multiple comparison post hoc test. ^∗^*p* < 0.05, ^∗∗^*p* < 0.01, and ^∗∗∗^*p* < 0.001. Bars with different letters are significant.

Serum TC and LDL-C contents were not significantly different between all groups tested (Figures 2(a) and 2(d)). However, coinfected subjects displayed significantly higher mean TG levels (219.88 ± 14.67 mg/dL vs. 191.82 ± 4.66  mg/dL, *p* < 0.05; Figure 2(b)) and lower HDL-C levels (9.05 ± 1.62 mg/dL vs. 18.93 ± 2.35 mg/dL, *p* < 0.05; Figure 2(c)), relative to patients with HEV-monoinfection. Moreover, there was no statistical difference between the mean HDL-C, TG, or LDL-C contents of the coinfected patients and the HBV-monoinfected group (Figures 2(b)–2(d)).

In the univariate analysis, HBV-monoinfected (82.76% vs. 35.3%, RR = 2.34, 95%CI = 1.206-4.559, *p* = 0.003) and HEV/HBV-coinfected subjects (88.89% vs. 35.3%, RR = 2.55, 95%CI = 1.297-5.014, *p* = 0.014) were associated with a higher risk of coronary heart disease than HEV-monoinfected patients as evidenced by CRI values (Table 4). However, none of the monoinfected groups was found to be considerably (*p* > 0.05) more exposed to liver injury than the coinfected group based on the percentage of people with elevated ALT (29.4%, HEV; 13.33%, HBV vs. 0%, HEV/HBV) and AST values (5.88%, HEV; 3.45%, HBV vs. 0%, HEV/HBV). This finding is consistent with the mean ALT (22.43 ± 4.34 IU/L vs. 33.32 ± 4.70 IU/L, *p* < 0.01; Figure 2(e)) and AST levels (12.38 ± 1.68 IU/L vs. 26.78 ± 2.19 IU/L, *p* < 0.001; Figure 2(f)) recorded in all groups, which remain in the normal range (<40 IU/L) and were found significantly lower in the coinfected group than in the HEV-monoinfected group.

**Figure 3:**
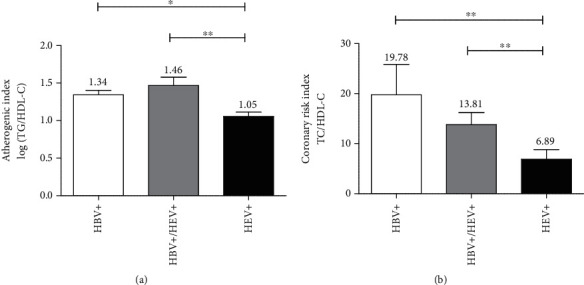
Mean coronary and atherogenic risk indexes of HBV/HEV-coinfected and monoinfected groups. The comparison was performed using the nonparametric Kruskal-Wallis test followed by Dunn's multiple comparison post hoc test. ^∗^*p* < 0.05, ^∗∗^*p* < 0.01.

As shown in Figure 3, the atherogenic indexes of all groups were greater than 0.5 (the maximum cutoff point set for normal individuals by previous studies [23]) and the values for the HBV-monoinfected group and the HEV/HBV-coinfected groups were significantly higher than those of the monoinfected HEV group (1.34 ± 0.06 and 1.46 ± 0.10, respectively, vs. 1.05 ± 0.05, *p* < 0.05) Similarly, the mean CRI values were considerably higher in both groups than in the HEV group (19.78 ± 6.06 and 13.81 ± 2.39, respectively, vs. 6.89 ± 1.93, *p* < 0.01), supporting the association observed in Table 4.

## 4. Discussion

In the current study, the serological analysis carried out on 179 participants revealed a prevalence of 21.22% of hepatitis B cases, including 16.2% in monoinfection. This result confirms the endemicity of HBV in Chad [25] and corroborates previous studies that reported a prevalence of 12.2% in control groups in N'Djamena [5]. For hepatitis E, the search for anti-HEV antibodies using the ELISA test revealed 20% of positive cases overall, including 12.59% in monoinfection with a strong percentage of IgG antibodies, suggesting a high frequency of an old infection. This result also confirms the endemicity of hepatitis E in Chad but remains lower than that found by Coursaget et al. in subjects surveyed in N'Djamena [7]. The observed discrepancy may be linked to the awareness of the population about hygiene practices. A survey carried out among households in the Am-Timan community made aware of hygiene measures after the 2016 epidemic showed that 99% of them used techniques such as water chlorination to limit the spread of hepatitis E infection [26].

To understand the reasons behind the circulation of viral hepatitis, a study was carried out on the risk factors involved in transmission. For this purpose, the statistical association between seropositivity and sociodemographic factors was analyzed.

For HBV infection, men were statistically more exposed than women. Likewise, subjects 33-39, 47-53, and 54-60 years old were also significantly more exposed than individuals [18–25] years old. This result is comparable with those of Khan et al., who reported a prevalence of 68.15% in men compared to 31.85% in women in Pakistan [27]. The gender disparity in HBV infection was demonstrated in earlier studies and is considered due to the effects of sex hormones. Androgen and estrogen hormones were shown to act oppositely on HBV transcription, immune response to HBV infection, and the progression of associated liver diseases [28].

Furthermore, participants in the second, fourth, fifth, and sixth arrondissements had a higher risk of getting hepatitis B than those in the eighth arrondissement, and this risk was significant for the 6th arrondissement. This could be explained by the poor vaccine coverage in these areas, which would prevent early collective protection. Indeed, in 2017, N'Djamena was cited among the four Chadian health regions in danger of epidemics due to inadequate vaccination coverage [29]. Very little data exist on vaccination coverage against hepatitis B in Chad in the literature. However, a recent WHO and UNICEF estimate puts the vaccine coverage rate at 58% in 2021 for this country, much below the 89% claimed by government officials [30]. In addition, the predominance of certain ethnic groups in these localities may have contributed to this exposure. Prior research conducted by Tchonchimbo et al. in the Lere Health Department (Chad) found that 95% of the 268 patients positive for hepatitis B, belonged to the Moundang tribe [31]. According to the authors, several cultural behaviors, unique to this group, enhance HBV transmission between families.

For hepatitis E, men were significantly more at risk than women. These results are consistent with those of Boonyai et al. [32] in Thailand (45.55% in men vs 36.53% in women), and Junaid et al. [35] in Plateau State, Nigeria (53.12% in men vs. 47.74% in women). This could be explained by the fact that in the Chadian society, as in many Asian and African societies, agropastoral activities are reserved mainly for men and constitute a source of exposure to hepatitis E, as demonstrated earlier [33]. However, Ataei et al. reported no significant difference between females and males (4.2% vs. 3.9%, *p* = 0.78) regarding HEV seroprevalence in a population-based study in the Isfahan Province, Iran [34]. The reason for this disparity could be related to differences in cultural practices. Likewise, the professional status was strongly connected to HEV seropositivity, farmers being the category most associated with hepatitis E. This result is close to that reported by Junaid et al. in Nigeria [35] who found a high anti-HEV IgG seroprevalence (66.4%) for farmers. According to these authors, HEV can be considered an occupational disease spread by close contact with domestic animals. In fact, Tritz et al. [33] in a recent study, reported that cattle farmers in Lao state had a significantly higher prevalence of anti-HEV IgG antibodies than a control group without cattle (59.1% vs. 43.1%, *p* = 0.008). Furthermore, a Chinese study reported a high seroprevalence of HEV in farmers of swine (49.1%) and cattle (26.5%), and among veterinarians (26.7%) and control (20.2%) subjects in Jilin province [36].

To our knowledge, very few studies have documented hepatitis B/E coinfection in Chad so far. In the current study, the results revealed a coinfection rate of 5% corroborating thereby previous work by Coursaget et al. in 1998 who found a 15% hepatitis B-hepatitis E coinfection rate in N'Djamena [7]. The analysis of the anti-HEV IgG titers between mono- and coinfected groups demonstrated that coinfected subjects have significantly lower IgG levels than monoinfected patients, suggesting thereby a decreased humoral response in the coinfected group. Both groups of patients were found to be asymptomatic, which means that their immune response is strong enough to protect against symptoms. However, for coinfected patients, the presence of HBV infection could have decreased the antibody response to HEV to the profit of HBV.

Previous studies have shown that subjects with viral hepatitis can also, in sporadic cases, be carriers of the dengue virus, resulting in a more severe deterioration of liver function [11, 12]. In this study, less than 2% of dengue cases were identified, including 0.83% of HEV/DENV cases. This very low incidence of dengue could be linked to several factors, in particular the low prevalence of dengue fever in the region, the study period not conducive to vector propagation, the target age group predominantly adult, while it was also shown that dengue fever circulates much more in children and adolescents than in adults [22].

Although this investigation discovered hepatitis B/E co-infection, the impact on cardiovascular risk remains unknown. The findings of this study revealed that the co-infected participants exhibited substantial hypertriglyceridemia, as well as lower HDL-C and LDL-C values than the HEV-monoinfected group. They were also shown to have a greater risk of developing dyslipidemia, with higher mean CRI and AI values than individuals infected with HEV-monoinfected. The role of LDL-C in the development of atherosclerosis, and therefore, cardiovascular disease is well documented [37] and is one of the top targets in cardiovascular therapy. While some authors argue for the sole use of this marker in atherogenic risk prediction [38], others suggest that a combination of lipids parameters and lipoproteins has the best predictive capacity as it provides more accurate data [24, 39]. In this regard, novel indicators such as AI and CRI have been proposed to improve the assessment of cardiovascular risk. AI, which is a logarithmic transformation of the TG/HDL-C ratio, was found to closely correlate with the size of the LDL particles as reported early [23]. While negative AI values are usually regarded as safe, and individuals from this category are seen as low risk for cardiovascular disease, people with positive values are generally presented as a higher risk group with LDL particles over 25 mm in diameter [23]. Since the AI values recorded in this study are positive, both the monoinfected and coinfected groups can be considered to be at high risk for atherogenic dyslipidemia and cardiovascular disease. These results are consistent with previous findings that reported the impact of HIV/HCV coinfection on lipid profile [18, 19]. The effect of HEV on lipid metabolism was little studied. Recent evidence suggests that HEV hijacks structures highly dependent on cholesterol, the so-called multivesicular bodies like the endosomal system for its release, reducing thereby the intracellular cholesterol level [40]. HEV was also found capable to alter cholesterol metabolism-related gene expression in cell culture. The role of hepatitis viruses coinfection on dyslipidemia has been preliminarily studied in previous works. In an American study, HCV/HIV coinfected patients had a significantly lower risk of hypercholesterolemia and hypertriglyceridemia than those HCV-monoinfected [18]. Similar findings were reported in subsequent studies targeting an Italian cohort [41] after initiation of highly active antiretroviral therapy.

Beyond this aspect of the lipid profile, the analyses also revealed that HEV/HBV-infected patients had a considerably decreased probability of developing liver damage than the monoinfected groups; however, this was not statistically significant. Furthermore, their mean transaminase levels were not considerably different from those of the HBV-monoinfected group but were much lower than those of the HEV-monoinfected subjects, indicating that coinfection appears to protect them from increasing liver damage compared to this group. These findings contradict earlier research, which revealed that the presence of HBV in people increased liver-related mortality. Indeed, Kilonzo et al. demonstrated that individuals with both HEV and chronic hepatitis B had higher ALTs (*p* < 0.001), total serum bilirubin (*p* < 0.001), and lower albumin levels (*p* < 0.001) than HBV-monoinfected patients in a retrospective study in Wuhan (China) [42]. Similar findings were also drawn from an early Chinese study conducted on patients with chronic hepatitis and superinfected by several hepatic viruses [43]. The authors found significantly higher ALT, AST, and total bilirubin content in the superinfected groups (HBV/HAV, HBV/HCV, and HBV/HEV) than in the monoinfected group with HBV. The discrepancy in our study might be explained by the lack of comparison with the HEV-monoinfected group or even by the fact that HBV infection is still early in the individuals and has not progressed to the point where it can cause problems.

## 5. Conclusions

In conclusion, the results of this study show that hepatitis B and E remain highly endemic in N'Djamena with a single prevalence of approximately 20% and a coinfection rate of 5%. Moreover, certain environmental and human conditions make some groups more exposed to these infections than others. HBV/HEV-coinfected patients were found to have a more reduced humoral response and a higher risk of developing coronary heart disease than HEV-monoinfected patients, partly due to HDL-hypocholesterolemia and high TG values. However, they remain asymptomatic. Due to the limited sample size of this study, some aspects of the practical significance of these data need further investigation. Therefore, larger clinical and mechanistic studies are required to confirm these effects and understand how this dyslipidemia process.

## Figures and Tables

**Table 1:** Distribution of participants according to the sociodemographic factors.

	Number	Percentage (%)
Sex		
Male	111	63
Female	68	37
Total	179	100
Age (years)		
18–25	46	26
26–32	47	26
33–39	31	17
40-46	20	11
47-53	16	9
54-60	12	7
61-70	07	4
Total	179	100
Professional situation		
Students	49	27
Housewife	41	23
Traders	26	15
Civil servants	27	15
Independents	31	17
Farmers	10	6
Total	179	100
Marital status		
Married	111	63
Single	68	37
Total	179	100
Educational level		
University	73	41
Secondary	37	21
Primary	47	26
Unschooled	22	12
Total	179	100
Knowledge of the HBV status		
Yes	26	15
No	153	85
Total	179	100
District of origin		
1st	14	8
2nd	06	3
3rd	12	7
4th	05	3
5th	08	4
6th	21	12
7th	39	22
8th	27	15
9th	18	10
10th	29	16
Total	179	100

**Table 2:** Effect of the sociodemographic factors on HBV seroprevalence.

	Positive	Negative	Relative risk	Confidence interval	*p* value
Gender					
Male	30	81	2.297	1.159-4.717	*0.0153* ^∗^
Female	8	60	1		
Age (years)					
18–25	3	43	1		
26–32	9	38	2.936	0.9269-9.621	0.1196
33–39	11	20	5.441	1.797-17.11	*0.0020* ^∗∗^
40-46	4	16	3.067	0.8192-11.28	0.1862
47-53	5	11	4.792	1.376-16.43	*0.0222* ^∗^
54-60	6	6	7.667	2.357-24.64	*0.0014* ^∗∗^
61-68	0	7	0.000	0.000-6.761	>0.9999
Professional situation					
Students	6	43	1		
Housewife	7	33	1.429	0.5410-3.775	0.5544
Traders	7	19	2.199	0.8442-5.648	0.1233
Civil servants	8	20	2.333	0.9252-5.858	0.1223
Independents	6	20	1.885	0.6924-5.021	0.3211
Farmers	4	6	3.267	1.096-8.701	0.0551
Marital status					
Married	28	83	1.715	0.9154–3.315	0.1313
Single	10	58	1		
Educational level					
University	13	60	1		
Secondary	9	28	1. 366	0.6431-2.814	0.4552
Primary	12	35	1.434	0.7197-2.822	0.3602
Unschooled	4	18	1.021	0.3699-2.567	>0.9999
Knowledge of the HBV status					
Yes	3	23	1		
No	35	118	0.5044	0.7488–5.901	0.2983
District of origin					
1st	2	12	1.929	0.3614–9.987	0.5956
2nd	2	4	4.500	0.8642-21.14	0.1422
3rd	2	10	2.250	0.4228-11.50	0.5733
4th	2	3	5.400	1.047-324.59	0.1053
5th	3	5	5.063	1.123-21.90	0.0665
6th	7	14	4.500	1.199-17.87	*0.0306* ^∗^
7th	9	30	3.115	0.8511-12.28	0.1772
8th	2	25	1		
9th	3	14	2.382	0.5141-11.04	0.3590
10th	4	25	1.862	0.4325-8.27	0.6708

Note: italic value indicates ^∗^*p* < 0.05; ^∗∗^*p* < 0.01 statistically significant.

**Table 3:** Effect of sociodemographic factors on HEV seroprevalence.

	Positive	Negative	Relative risk	Confidence interval	*p* value
Sex					
Male	24	62	4.558	1.591-13.84	*0.0018* ^∗∗^
Female	3	46	1		
Age (years)					
18–25	6	22	1.286	0.4787–3.422	0.7502
26–32	6	30	1		
33–39	6	20	1.385	0.5169-3.664	0.5364
40-46	3	14	1.059	0.3107-3.347	>0.9999
47-53	3	10	1.385	0.4099-4.227	0.6831
54-60	2	8	1.200	0.2945-4.175	>0.9999
61-68	1	4	1.200	0.1981-5.165	>0.9999
Professional situation					
Students	10	28	8.421	1.533–49.92	*0.0087* ^∗∗^
Housewife	1	31	1		
Traders	4	12	8.000	1.301-50.68	*0.0366* ^∗^
Civil servants	4	19	5.565	0.8982-35.79	0.1490
Independents	5	14	8.421	1.420-52.22	*0.0222* ^∗^
Farmers	3	4	13.71	2.151-86.62	*0.0140* ^∗^
Marital status					
Married	15	71	1		
Single	12	37	1.404	0.7168-2.701	0.3736
Educational level					
University	15	43	1.940	0.7665–5.281	0.2742
Secondary	8	23	1.935	0.6945-5.595	0.3354
Primary	4	26	1		
Unschooled	0	16	0.000	0.000–1.598	0.2820
District of origin					
1st	2	11	1.436	0.3051–6.343	0.6448
2nd	2	2	4.667	1.045-16.78	0.1053
3rd	1	3	2.333	0.3525-11.62	0.4306
4th	0	3	0.000	0.000-7.578	>0.9999
5th	0	6	0.000	0.000–4.637	>0.9999
6th	6	10	3.500	1.089-11.49	0.0534
7th	3	25	1		
8th	3	19	1.273	0.3155-5.084	>0.9999
9th	4	10	2.667	0.7395-9.465	0.1967
10th	5	18	2.029	0.5911-7.097	0.4419

Note: italic value indicates ^∗^*p* < 0.05; ^∗∗^*p* < 0.01 statistically significant.

**Table 4:** Statistical association between coronary risk index frequencies, liver alteration (expressed as high transaminase values), and the mono- and coinfection status.

Parameters	Variables	HBV group (*n* = 29)	HBV/HEV group (*n* = 9)	HEV+ group (*n* = 17)
CRI (TC/HDL-C)	High (>4.85)	24 (82.76%)	8 (88.89%)	6 (35.3%)
	Low (<4.85)	5 (17.24%)	1 (11.11%)	11 (64.70%)
	Relative risk	2.34	2.55	1
	95% CI	1.206-4.559	1.297-5.014	
	*p* value	0.003^∗∗^	0.014^∗^	

ALT	High (>41 IU/L)	4 (13.33%)	—	5 (29.4%)
	Normal (<41 IU/L)	25 (86.67%)	9 (100%)	12 (70.6%)
	95% CI	—		—
	Relative risk		1	
	*p* value	0.55		0.128

AST	High (>40 IU/L)	1 (3.45%)	—	1 (5.88%)
	Normal (<40 IU/L)	28 (96.55%)	9 (100%)	16 (94.12%)
	Relative risk		1	—
	95% CI	—		—
	*p* value	1		1

Statistical association was analyzed using the Fisher's exact test. ^∗^*p* < 0.05, ^∗∗^*p* < 0.01.

## Data Availability

Data of this manuscript are available upon reasonable request to the corresponding author.
